# Electroluminochromic Materials: From Molecules to Polymers

**DOI:** 10.3390/polym11010098

**Published:** 2019-01-08

**Authors:** Ming Hui Chua, Qiang Zhu, Kwok Wei Shah, Jianwei Xu

**Affiliations:** 1 Institute of Materials Research and Engineering (IMRE), Agency for Science, Technology and Research (A*STAR), 2 Fusionopolis Way, Innovis, #08-03, Singapore 138634, Singapore; chua_ming_hui@imre.a-star.edu.sg (M.H.C.); zhuq@imre.a-star.edu.sg (Q.Z.); 2 Department of Building, School of Design and Environment, National University of Singapore, 4 Architecture Drive, Singapore 117566, Singapore

**Keywords:** electroluminochromic, electrofluorochromic, electrochromic, polymers, metal complexes

## Abstract

Electroluminochromism is an interesting property found in certain classes of molecules and polymers whose photoluminescence can be modulated through the application of an external electrical bias. Unlike electrochromic materials, electroluminochromic counterparts and their applications are comparatively fewer in quantity and are less established. Nonetheless, there prevails an increasing interest in this class of electro-active materials due to their potential applications in optoelectronics, such as smart-displays, and chemical and biological sensing. This review seeks to showcase the different classes of electroluminochromic materials with focus on (i) organic molecules, (ii) transition metal complexes, and (iii) organic polymers. The mechanisms and electroluminochromic performance of these classes of materials are summarized. This review should allow scientists to have a better and deeper understanding of materials design strategies and, more importantly, structure-property relationships and, thus, develops electroluminochromic materials with desired performance in the future.

## 1. Introduction to Electroluminochromism

The optical properties of electro-active materials can often be modulated via the application of an external electrical bias. Electrochromism is a phenomenon displayed by some materials of exhibiting reversible changes in physical colour upon electrically-induced oxidation and/or reduction, whereas when materials display reversible changes in photoluminescence whether emission intensity or colour, it is said to be electroluminochromism. Most electroluminochromic (ELC) materials involve changes in fluorescence properties and, hence, the term electrofluorochromic (EFC) can be used interchangeably. However, there are some materials emitting phosphorescence and, thus, the more general term “electroluminochromism” is used to encompass the modulation of both types of phenomena. Over the past two decades, there has been intense research and a plethora of studies on electrochromic (EC) materials due to their highly translatable applications, such as smart windows, smart optoelectronic displays, military camouflage coatings, etc., some of which have already been commercialized [1,2,3]. On the other hand, research on ELC materials appears to receive less attention and interest in comparison with EC materials. Likewise, ELC materials are also particularly useful in optoelectronics applications, like smart displays. In addition, the possibility to modulate luminescence via an electrochemical way also makes ELC materials largely applicable for sensing applications.

Similar to EC devices, a typical preliminary ELC device consists of electroactive ELC materials sandwiched between two transparent ITO-coated plates, in the presence of another layer of gel electrolyte in between. Due to the scope of this review, aspects of ELC device fabrication and its performance optimization will not be included. Like most optoelectronic devices, the ELC materials are casted as thin films and, most importantly, their film emission behaviour in general differs from that in the solution. Firstly, red- or blue-shifting of emission may be observed due to different molecular packing of the often highly-crystalline ELC materials: the formation of J-aggregates leads to red-shifting of emission, whereas the formation of H-aggregates results in the blue-shifting [4,5,6]. Secondly, most conventional structurally planar organic luminogens are subjected to aggregation-caused quenching (ACQ) due to the formation of excimers or exciplexes which are attributable to the formation of favourable intermolecular π-π interactions [7]. As such, these luminogens exhibit little or no emission in the thin film state even though they emit brightly in their dilute solution, which is undesirable for thin-film ELC device performance. The aggregation-induced emission (AIE) and aggregation-induced enhanced emission (AIEE) luminogens discovered in the year 2001, however, overcame this thorny issue of ACQ, thus allowing optoelectronic materials to emit brightly even in the thin film state [8,9,10,11]. Therefore, the AIE property has been incorporated in the molecular design of many organic ELC materials, which will be subsequently summarized in this review. 

Measurements of photoluminescence spectra of ELC thin films can be preliminarily performed by placing a three-electrode-connected device at 45° to the incident excitation light source in a typical photoluminescence spectrophotometer. Determination of quantum yield or quantum efficiency (ϕ_f_), however, requires the use of an integrating sphere. Apart from the original colour and quantum yield/efficiency of emission, key ELC performance parameters include (i) contrast ratio (I_OFF/ON_), particularly for materials whose photoluminescence is quenched or weakened upon applying an external voltage; (ii) response times, i.e., the time taken to completely “switch on and off” the photoluminescence of the material or device; as well as (iii) stability, determined by loss of the original photoluminescence intensity after a certain number of switching cycles. Additionally, in many cases of studies on organic ELC molecules and polymers, observations were made to confirm the absence of material decomposition or the formation of unwanted side products due to no shifting of emission maxima, λ_max_, during photoluminescence switching. 

Over the years, researchers have developed quite a sizable number of compounds exhibiting electroluminescence properties. These include organic molecules, polymers, transition metals and lanthanide complexes, polyoxometalates (POM), as well as quantum dots. The mechanisms responsible for ELC properties differ from each class of materials, which will be subsequently reviewed in great detail. To date, several reviews in the field of electroluminochromism are already available, providing scientists a broad coverage of different aspects from ELC mechanisms to device fabrication and optimization [12,13,14,15]. In this review, we seek to showcase the structural diversity of many different reported ELC materials, with focus on the following categories: (i) organic molecules, including dyads and redox-active molecules; (ii) transition metal complexes; and (iii) organic polymers, including conjugated and non-conjugated polymers. Through various examples in each category and sub-category, different ELC mechanisms, as well as ELC performance parameters, will be summarized. It is envisaged that through the structure-performance comparisons provided in this review, a deep understanding could be developed towards the rationale behind structural designs of different ELC materials, which can be taken into consideration in the development of future ELC materials with desirable properties and better performances.

## 2. Organic Small ELC Molecules

Organic small molecules with ELC properties can be generally classified as either dyads or redox/electro-active fluorophores. In the former category, organic fluorophores are attached to a redox-active moiety, such as tetrazine, ferrocene, quinone, and tetrathiafulvalene (TTF), either by π-conjugated bridging groups, or tethered with saturated hydrocarbon chains. By internal electron or energy transfer, the electro-active group affects the fluorescence intensity of the fluorophore it was attached to and, hence, the entire dyad molecule. As such, by applying an external electrical bias, the electro-active moiety can be reversibly reduced or oxidised, either enabling or prohibiting internal electron transfer (such as photoinduced electron transfer, PET), or energy transfer (such as Förster resonance energy transfer, FRET) between itself and the fluorophore, hence leading to the modulation of the dyad’s fluorescence intensity. On the other hand, for the latter category of redox/electro-active fluorophore, the organic molecule on its whole is able to undergo reversible oxidation and/or reduction upon the application of an external electrical bias, thus forming radical ion species which are often weakly or non-fluorescent. There are also examples where, although rare, fluorophores undergo a change in fluorescence colour upon reduction or oxidation. This allows the modulation of fluorescence properties by switching between the neutral and radical-ion states. 

**Figure 1:**
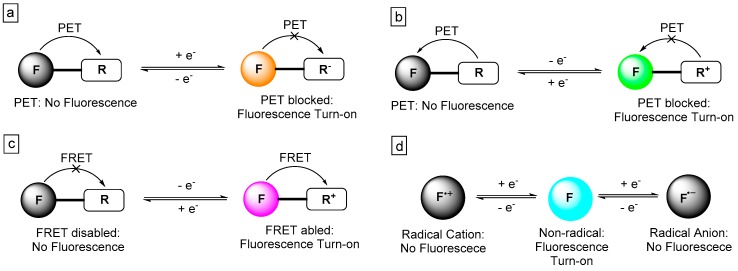
General mechanisms of electroluminochromism in organic small molecules: Dyad systems involving internal electron transfers (**a**,**b**), and internal energy transfer (**c**) between fluorophore, F, and electroactive moiety, R, attached; as well as systems involving electroactive fluorophore (**d**) that fluorescence quenches on being oxidised/reduced to radical species.

Figure 1 summarises the different general mechanisms of electroluminochromism for the two categories of ELC organic small molecules. The first three cases (a–c) illustrate plausible mechanisms of dyads-based ELC materials. A fluorophore, F, may be attached to an electroactive moiety, R, as reflected in (a) and (b), respectively. The excited electron from the photo-excited fluorophore may transfer into the LUMO of electron-poor R, whereas an electron from the HOMO of an electron-rich R may transfer into the singly-occupied original HOMO of the photo-excited fluorophore, leading to quenching of fluorescence. Application of an external electrical bias can result in the reduction or oxidation of R groups for (a) and (b), thus rendering them incapable of accepting or donating electrons to and from the excited fluorophore, respectively. The deactivation of the PET process thus results in fluorescence turn-on. Similarly in the case of (c), the application of an external potential bias may shift the absorption profile of the electro-active moiety, R, towards or away from overlapping with the emission profile of the fluorophore. The enabling and disabling of FRET process can, thus, be used to modulate the fluorescence of the ELC system. Finally, the mechanism for the redox/electro-active fluorophore ELC system can be represented by (d). In the neutral state, these fluorophores emit light. When an external electrical bias is applied to the redox/electro-active fluorophore, they will be oxidised or reduced into radical cations or radical anions, making them non-fluorescent.

### 2.1. ELC Dyads

**Figure 2:**
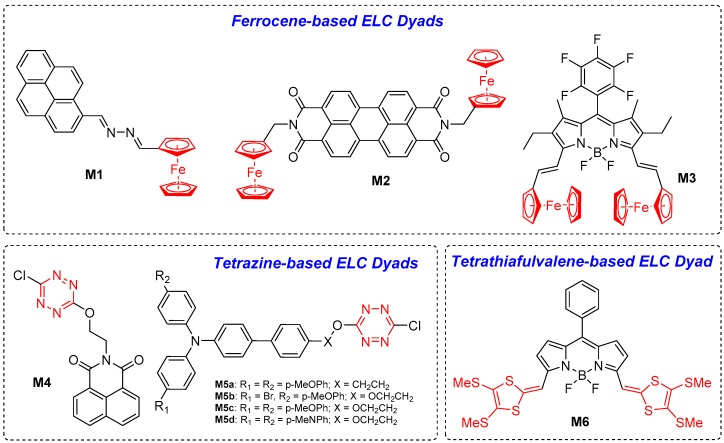
Chemical structures of ELC molecular dyads.

Dyads consist of organic fluorophore structurally attached to one or more redox-active moieties. As shown in Figure 1, a reduction or oxidation of the redox-active moiety will modulate the photoluminescence of the organic fluorophore via introduction or removal of electron transfer processes (such as PET or FRET), or simply via the fluorescence quenching via radical species. In this sub-section, several ELC dyads consisting of redox-active ferrocene, tetrazine, and tetrathiafulvalene will be discussed. The chemical structures of these dyads are shown in Figure 2 and their key ELC performance parameters are summarised in Table 1. 

#### 2.1.1. Ferrocence-Based ELC Dyads

Ferrocene is a well-known and widely studied electroactive group which is commonly used as a reference standard for electrochemical analysis. It would, thus, be reasonable to incorporate ferrocene in the structural designs of ELC dyads. Veciana et al. first reported dyad **M1** with a ferrocene unit attached to pyrene fluorophore via a 2,3-diaza-1,3-butadiene bridge [16]. A solution of **M1** in the neutral state exhibited weak fluorescence with an emission maximum (λ_em_) of 385, 405, and 425 nm and a low ϕ_f_ of 0.0035, which was attributed to electron and energy transfer from the electron-donating ferrocene to the excited state of pyrene. Fast and reversible oxidation of **M1** reduces electron-donating ability of the ferrocene, thus limiting the transfer processes and, hence, leading to fluorescence enhancement [16]. Similarly, Yao et al. reported molecule **M2** in which perylene diimide is attached to two ferrocene units at the *N*- positions via methylene linkages [17]. The PET process between the perylene diimide fluorophore and ferrocene moieties in the neutral state results in weak fluorescence, but the reversible oxidation of the ferrocene to form a radical cationic species inhibits the process, causing significant fluorescence enhancement at λ_em_ = 533 nm. Miomandre et al. reported the boron-dipyrromethene (BODIPY)–ferrocene dyad **M3** in which the ferrocene groups were attached to the BODIPY core at the 3,5- positions via an ethylene bridge [18]. Fluorescence of BODIPY in **M3** is quenched in the neutral state due to PET, but bielectronic oxidation of the ferrocene groups into the ferrocenium state inhibits PET and subsequently causes fluorescence amplification. Chronoamperometric and electro-switching studies for all three dyads confirm the reversibility of fluorescence switching between stepwise oxidation and reduction processes. 

#### 2.1.2. Tetrazine-Based ELC Dyads

Tetrazine is another electroactive moiety that is used to develop ELC dyads. Audebert et al. previously reported a series of highly fluorescent tetrazine derivatives that can undergo reversible fluorescence quenching when the tetrazine moiety is electrochemically reduced to the radical anionic form [19]. On the basis of this, Kim and Audebert et al. developed dyad **M4** as a fluorescent switch where chlorotetrazine was tethered to perylene imide fluorophore via an ethoxy-linkage at the *N*- position [20]. Three different states can exist in dyad **M4**: a neutral state, a tetrazine reduced state, and both a tetrazine and naphthalene imide reduced state. Dyad **M4** fabricated on a solid state cell exhibited vivid yellow fluorescence (attributed to tetrazine) at positive or no applied potentials but the fluorescence was diminished and eventually quenched when negative potentials up to −1.4 V were applied [20]. It was noted that there was no shift in spectral band and blue fluorescence from naphthalene imide was never observed even in the tetrazine-reduced states. Following this, a series of triphenylamine-tetrazine dyads **M5a**–**d** were subsequently reported by Audebert et al., in which differently-substituted triphenylamine moieties were attached to chlorotetrazine via 4-ethoxylphenyl or 4-ethylphenyl bridges [21]. Compounds **M5a**–**d** were non-fluorescent in the neutral state due to PET from triphenylamine unit to tetrazine moiety. One-electron oxidation of triphenylamine units to the radical cationic state inhibits the PET process, thus leading to fluorescence turn-on of the tetrazine unit. 

#### 2.1.3. Tetrathiafulvalene-Based ELC Dyad

Tetrathiafulvalene-BODIPY compound **M6** was examined to show both EC and EFC behaviours [22]. Stepwise oxidation of **M6** first gave a radical cationic state at one TTF moiety to lead to the quenching of the fluorescence with a λ_max_ of 803 nm, and further oxidation to form a non-radical dicationic state, in which both TTF moieties were oxidised, caused the emergence of a new fluorescence signal at λ_max_ = 1185 nm. This allows redox-switchable **M6** to be fabricated to an EFC device, which is able to modulate fluorescence in the near infrared (NIR) region on an unprecedented “on-off-on” regime [22].

#### 2.1.4. Redox-Active Moiety—Fluorophore Mixed System

Interestingly, it is not necessary for redox-active moieties to be attached to fluorophores in the form of a dyad to enable an ELC system. Such a “mixed-component ELC system” consists of one or more luminescent compounds and a redox-active species, which is able to modulate the fluorescence property of the luminescent component upon applying an external potential bias. Kobayashi et al. first reported an ELC system comprising redox-active diheptyl viologen, and luminescent Eu(hfa)_3_(H_2_O)_2_ complex, whereby intense red luminescence at 615 nm can be reversibly quenched as the external voltage applied increased from 0 to 2.2 V [23]. Zhang et al. subsequently reported an ELC system involving redox-active p-benzoquinone and highly fluorescent fluorescein dye [24]. In this system, no emission was observed but yellow fluorescence (λ_em_ = 535 nm) emerged when a negative potential bias of −1.5 V was applied, and this device exhibited outstanding stability and reversibility of over 1500 cycles [24].

**Figure 3:**
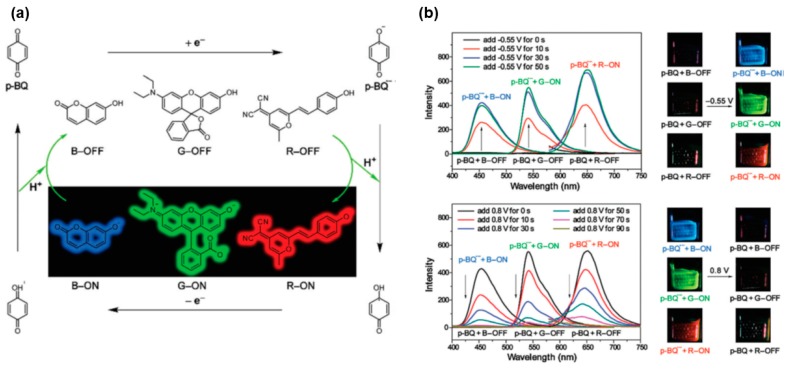
(**a**) Fluorescence switching mechanism of p-benzoquinone-mediated RBG EFC system. (**b**) Fluorescence spectra and photo of working electrodes under UV irradiation of mixture of p-benzoquinone with the three individual fluorophores, on the application of external negative and positive electrical bias. Reproduced with permission from [25]. Copyright 2017, the Royal Society of Chemistry.

More recently, Zhang et al. devised an pH-sensitive EFC system comprising a redox active p-benzoquinone moiety acting as an electro-base, 7-hydroxycoumarin, rhodol and 2-(2-(4-hydroxystyryl)-6-methyl-4*H*-pyran-4-ylidene)malononitrile to give pH-controllable blue, green, and red fluorescence, respectively [25]. Figure 3a shows the fluorescence switching mechanism of this EFC system by making use of the reversible acid/base-mediated redox property of p-benzoquinone, and the base-sensitivity of the three fluorophores. The EFC device fabricated from this system enables the fast and reversible modulation of red-blue-green (RBG)-coloured fluorescence via applying an external electrical bias to switch on or off the fluorescence (Figure 3b), making the device potentially useful for encrypted information storage and display applications [25]. 

### 2.2. Electroactive and Redox-Active Fluorophores

**Figure 4:**
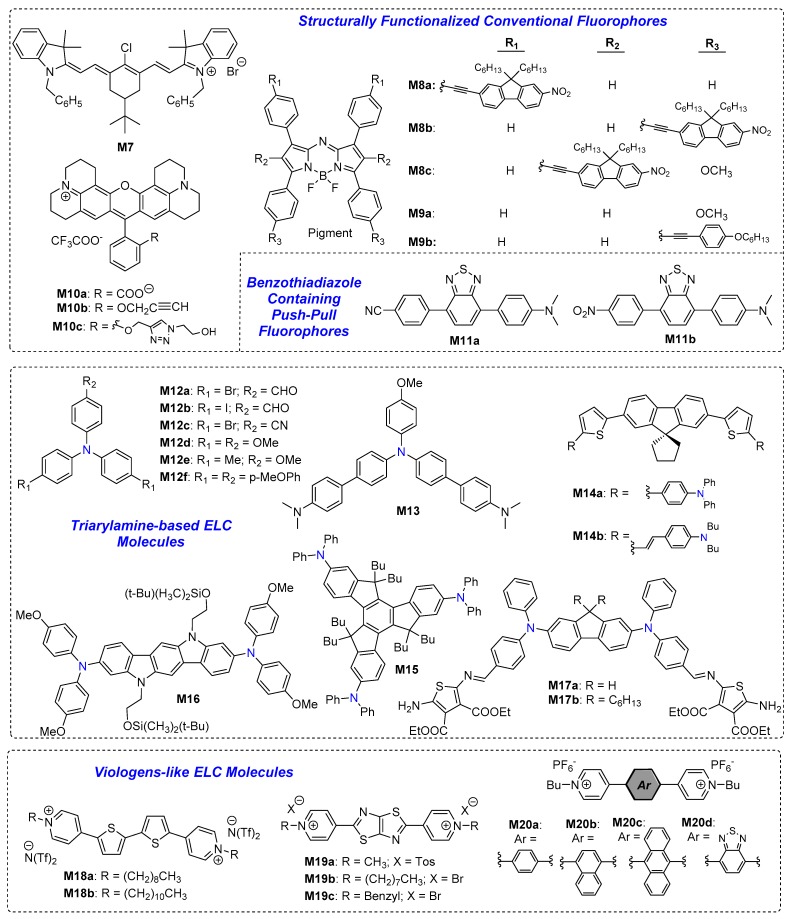
Chemical structures of ELC electroactive organic molecular fluorophores.

Unlike dyads where fluorescence quenching or enhancement involves energy or electron transfer processes, some electroactive molecules are able to modulate their fluorescence properties via electrochemical oxidation and/or reduction, thereby switching between neutral and radical ionic forms. These include some viologen derivatives as well as arylamine-based compounds. For the former, viologens-based liquid crystals have been reported to be a good candidate for EFC optical displays applications, whereas for the latter, a common structural similarity is the presence of at least two sp^2^-hybridised nitrogen atoms in conjugation across two ends of the molecule, separated by π-conjugated groups in between (e.g., cyanine structure). Figure 4 below shows the chemical structures of some key examples of electroactive organic molecular EFC fluorophores. We herein highlight these key examples of ELC fluorophores based on the following three categories: (i) conventional fluorophores that were structurally functionalized, (ii) triarylamine-based systems, and (iii) viologen-like derivatives. The ELC performance parameters of these examples are summarized in Table 2. 

#### 2.2.1. Structurally Functionalized Conventional Fluorophores

Some conventional fluorophores were reported to exhibit ELC properties. These fluorophores often contain two nitrogen atoms in conjugation with each other, which can be described as a cyanine system, making them redox-active. 

**Figure 5:**
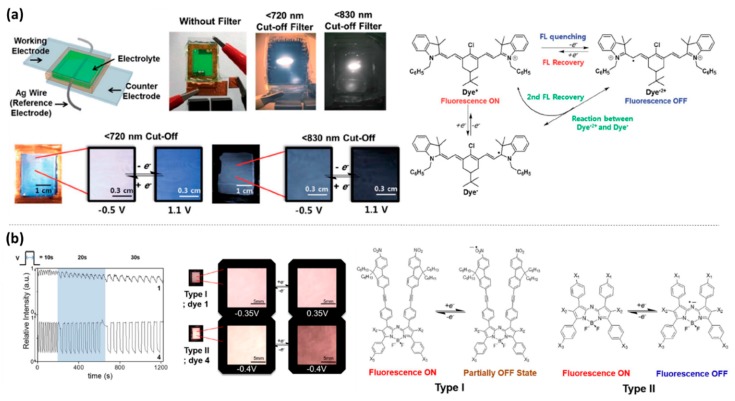
(**a**) White light emitting EFC device fabricated from **M7** and mechanism of fluorescence switching of **M7**. Reproduced with permission form [26]. Copyright 2014, The Royal Society of Chemistry. (**b**) EFC switching properties of **M8a** (denoted **1**) and **M9a** (denoted **4**) with corresponding NIR images attached. Also shown is the mechanism of fluorescence switching of the aza-BODIPY dyes. Reproduced with permission from [27]. Copyright 2016, Nature Publishing Group.

To begin with, Kim et al. reported a NIR emissive cyanine dye **M7** which is able to undergo reversible fluorescence switching between applied voltages of −0.5 and 1.1 V (vs. Ag wire) [26]. Electrochemical oxidation of mono-cationic **M7** resulted in a decrease in fluorescence intensity of 820 nm without any peak shifts due to the formation of radical dication as shown in Figure 5a. On the other hand, electrochemical reduction of **M7** led to the formation of the neutral radical dye. **M7** was fabricated into an EFC device, emitting white light with over 1000 on-off cycles (Figure 5a) [26].

Kim et al. also reported a series of NIR EFC aza-BODIPY dyes, **M8a**–**c** and **M9a**–**b** [27]. **M8a**–**c** contains nitrofluorene ethynyl units substituted at three different positions of the aza-BODIPY core, whereas **M9a**–**b** contain 4-methoxyphenyl and (4-(hexyloxy)phenyl)ethynyl groups substituted at the 3,5-positions, respectively. EFC studies demonstrated that all five dyes exhibited reversible fluorescence switching upon one-electron oxidation, with radicals stabilised over the nitro groups and over *meso*- nitrogen atom, for cationic species of **M8a**–**c** and **M9a**–**b**, respectively. The fluorescence of **M8a**–**c** (Type I) with nitro groups could not be fully quenched upon oxidation, leading to a lower fluorescence contrast between neutral and oxidised states. On the other hand, **M9a**–**b** (Type II) containing alkoxylphenyl groups display a much higher switching contrast with an I_ON/OFF_ ratio of 6.1 and more than 1000 on-off cycles [27]. The mechanisms of fluorescence switching for both Type I and II of aza-BODIPY dyes are as shown in Figure 5b. 

More recently, Buriez et al. reported electrochemical fluorescence switching of three rhodamine derivatives **M10a**–**c** [28]. Compounds **M10a**, **b**, and **c** have λ_em_ at 593, 614, and 600 nm, respectively, but one-electron electrochemical reduction of the three compounds into the radical neutral form resulted in fluorescence quenching. The fluorescence switching is completely reversible and the synthesis of **M10c** from **M10b** via Click chemistry opens the opportunity for the rhodamine system to be used as redox-active fluorescence probes especially in fluorescence confocal microscopy [28].

#### 2.2.2. Benzothiadiazole Containing Push-Pull Fluorophore

Skene et al. reported two push-pull *N,N*-dimethylamino-benzothiadiazole fluorophores, **M11a** and **M11b**, which were found to exhibit solvochchromism, electrochromic and electrofluorochromic properties [29]. Thin film of **M11a** with a terminal cyano group changed visible colour from yellow to mauve upon oxidation, with fluorescence colour correspondingly changing from purple to blue. On the other hand, thin film of **M11b** with a terminal nitro group exhibited a colour change from yellow to pale violet, and a fluorescence colour change from grey-green to green, upon undergoing oxidation [29].

#### 2.2.3. Triarylamine-Based ELC Molecules

**Figure 6:**
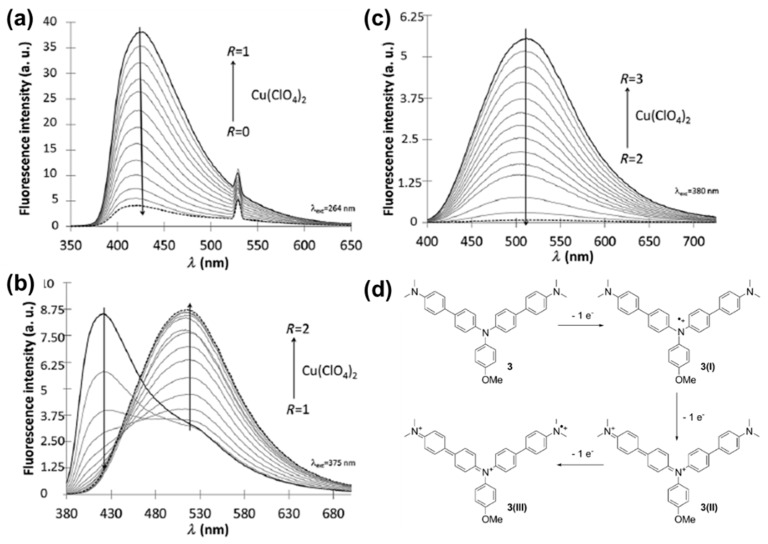
Fluorescence spectra changes of **M13** upon undergoing successive step-wise oxidation (with Cu(ClO_4_)_2_ as oxidant) from neutral to +1 state (**a**); +1 to +2 state (**b**); and +2 to +3 state (**c**). The mechanism of stepwise oxidation is as shown in (**d**). Reproduced with permission from [32]. Copyright 2015, Wiley-VCH Verlag GmbH and Co. KGaA, Weinheim.

Triphenylamine is a well-known building block for EC materials given that it is able to form stable radical cations at the nitrogen atom upon oxidation [30]. Miomandre and Alain-Rizzo et al. studied the effects of substituents on triphenylamine’s phenyl rings on EFC properties of a series of compounds **M12a**–**f** [31]. In the neutral state, the six derivatives emit fluorescence in the blue to green region and the generation of triphenylaminium radical cation via reversible electrochemical oxidation effectively quenches their fluorescence. Through the structure-performance relationship studies, it was found that triphenylamine derivatives with electron-donating substituents were preferred due to the generation of more stable radical cations on oxidation [31]. Following this, Miomandre and Alain-Rizzo et al. reported triphenylamine-based molecule **M13** functionalized with *N,N*-dimethylaniline on two of the phenyl groups [32]. Due to the presence of three nitrogen atoms, **M13** was found to exhibit four stable redox states (Figure 6d) and three protonated states, resulting in changes in colour and emission upon changing from one state to another. Neutral **M13** gave fluorescence with a maximum of 424 nm. On undergoing the first oxidation from neutral to the radical mono-cationic form, dramatic fluorescence quenching was observed (Figure 6a). Quenching of 424 nm peak was slowed down upon undergoing the second oxidation to the non-radical dicationic form, with the appearance of a new emission band peaking at 513 nm, which was effectively quenched upon undergoing the third oxidation to the radical tricationic form (Figure 6b,c) [32]. 

**Figure 7:**
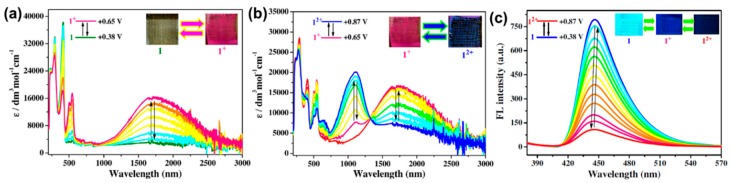
Electrochromic properties of **M16** with colour changes upon undergoing first (**a**) and second oxidation (**b**); as well as EFC properties of **M16** (**c**) upon undergoing stepwise oxidation. Reproduced with permission from [34]. Copyright 2017, Elsevier B. V.

Blanchard-Desce and Sojic et al. developed a series of three fluorene-based electrochemiluminescent dyes **M14a**, **M14b**, and **M15**, with λ_em_ at 496, 546, and 388 nm, and φ_f_ of 0.71, 0.67, and 0.40, respectively [33]. Amongst these, **M14a** generates electrochemiluminescence with the highest ϕ_f_ of 4.54 times that of [Ru(bpy)_3_]^2+^ reference upon undergoing reversible oxidation, attributing to good stability of its oxidised states [33]. More recently, Liu et al. reported indolo[3,2-b]carbazole-based molecule **M16** with two redox-active di(p-methoyphenyl)amine groups [34]. **M16** could be reversibly oxidised stepwise changing from light yellow to red then to blue, as shown in Figure 7a,b, whereas its bright blue emission (λ_em_ = 446 nm) gradually weakened upon first oxidation to mono-cationic state, and was completely quenched upon subsequent oxidation to the dicationic state, as shown in Figure 7c.

Finally, Skene et al. developed two azomethines molecules, **M17a** and **M17b**, containing a central fluorene core conjugated with *N*,*N*-diphenylamino groups at the 2,7-positions [35]. Both molecules exhibited solvochromism and were also found to be AIE-active. Upon an external oxidative potential, **M17a** and **M17b** thin films in poly(methacrylate) matrix changed from yellow to blue, whereas the red fluorescence of the aggregates of both compounds (in THF/water 1:9 *v*/*v*) was quenched [35].

#### 2.2.4. Viologen-Like ELC Molecules

**Figure 8:**
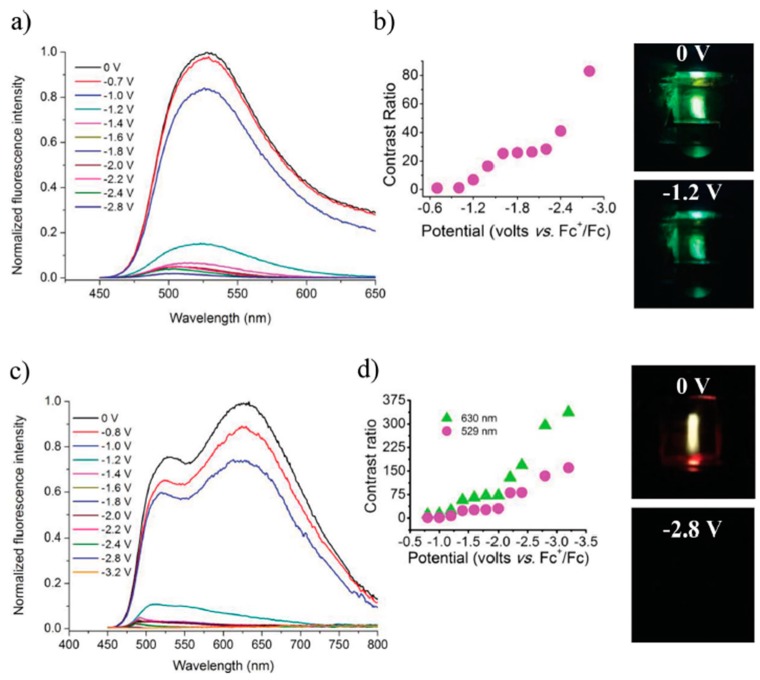
EFC properties of gel: Fluorescence spectra of EFC gel with 2% *w*/*w* (**a**) and 10% *w*/*w* (**c**) loading of **M18a** under different applied voltages; Corresponding contrast ratio of EFC gel with 2% *w*/*w* (**b**) and 10% *w*/*w* (**d**) loading of **M18a** versus different applied potentials, with photos of the respective EFC gel devices under different applied voltages as shown. Reproduced with permission from [37]. Copyright 2015, Wiley-VCH Verlag GmbH and Co. KGaA, Weinheim.

Viologens are classical EC materials which undergo reversible electrochemical oxidation into neutral resonance structures of both bipolarons and radical forms [3]. It would, thus, be reasonable to explore their derivatives for the development of EFC materials. Beneduci et al. first reported two thienoviologens’ columnar EFC liquid crystals **M18a** and **M18b** in different mesophases [36]. Electro-reduction of **M18a**–**b** induced the formation of neutral radical thienoviologen, resulting in an enhancement in red fluorescence. A fluorescence intensity contrast of up to 79% was recorded for **M18b** and both liquid crystals displayed a fast switching speed of <3.5 s due to efficient electron transport via π–π interactions facilitated by the liquid crystal packing [36]. Beneduci et al. subsequently incorporated **M18a** at low loadings of 2 to 10% *w*/*w* into polyvinyl formal (PVF) polymer matrix to afford a highly fluorescent EFC gel (ϕ_f_ up to 67%), which was sandwiched between two ITO electrodes in the fabrication of an EFC device [37]. At a higher loading, a second λ_em_ at 630 nm emerged due to molecular aggregation and, hence, emission colour differed as well. As shown in Figure 8, electro-reduction of the device with 2 wt % **M18a** resulted in lower green emission in intensity with an I_OFF/ON_ ratio of 87, whereas for device with 10 wt % **M18b**, total fluorescence quenching was observed with an I_OFF/ON_ ratio of up to 337 [37].

Walter et al. reported the three thiazolo[5,4-d]thiazole-based viologens derivatives **M19a**–**c** with different alkyl groups attached at the viologens’ *N*- positions [38]. The thiazolothiazole-based viologens exhibited intense blue fluorescence in the neutral state with ϕ_f_ between 0.8–0.96 due to their highly planar structure. On successive electrochemical reductions, however, the blue fluorescence became less intense due to the formation of mono- and bi- radical species [38]. More recently, Xu et al. reported four novel viologen derivatives with EC and EFC properties, **M20a**–**d**, with phenyl, naphthyl anthracenyl, and benzothiadiazole groups, respectively, between the two pyridinium units [39]. Reduction of four viologen derivatives (achieved at applied potentials between −1.4 V to −3.1 V) resulted in change in colour from bleached to coloured state. This was accompanied by partial quenching of blue, brilliant blue, yellow, and blue fluorescence exhibited by **M20a**–**d**, respectively, due to the formation of highly stable radical species [39].

## 3. ELC Transition Metal Complexes

**Figure 9:**
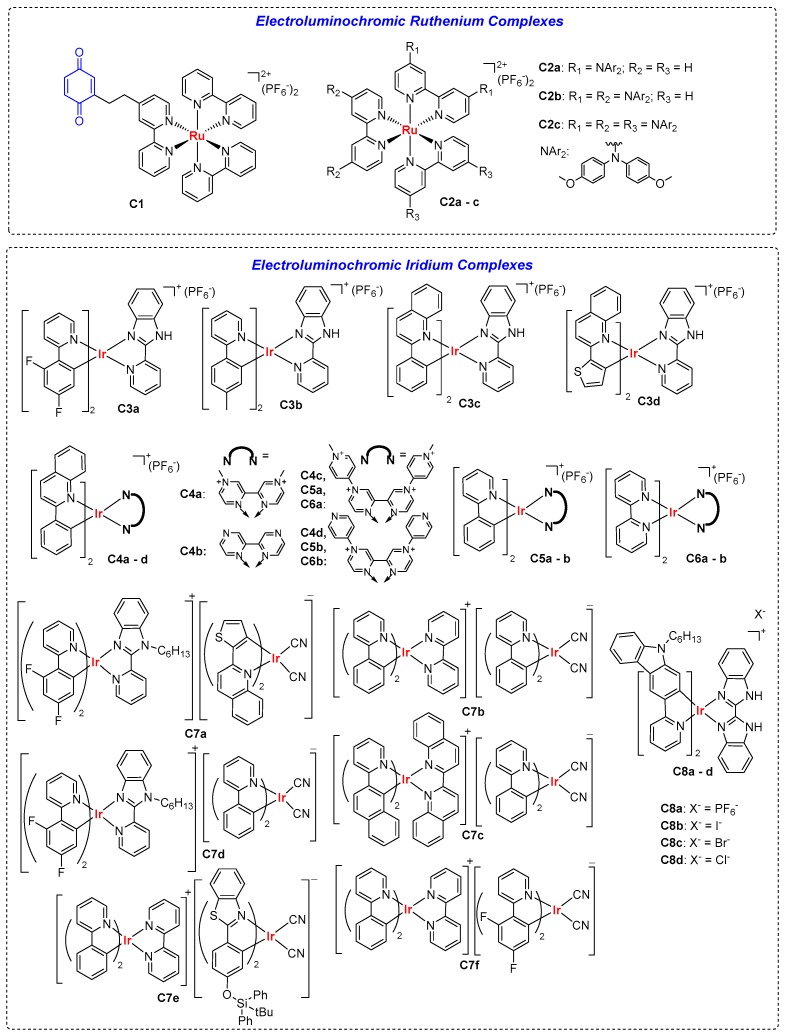
Chemical structures of transition metal complexes with ELC properties.

Like organic molecules, luminescent transition metal (TM) complexes can be functionalized to act as novel EC and ELC materials. Figure 9 shows the chemical structures of some typical examples with their ELC parameters summarised in Table 3. Luminescent TM complexes often contain metal centres with d^6^, d^8^, and d^10^ electronic configurations, for instances, iridium and ruthenium [40]. The coordinated ligands also affect photoluminescence properties as well via metal–ligand interactions. Many TM complexes are electroactive often at the metal centres, making them ideal candidates for EC and ELC materials. Most of the examples discussed herein involve the luminescence-switching of electroactive emissive TM complexes, though there is also an example involving emissive TM complex tethered to a redox-active group in the form of a dyad. In addition, TM complexes may also emit longer lifetime phosphorescence, as compared to organic fluorophores which mostly emit fluorescence.

### 3.1. ELC Ruthenium Complexes

Ruthenium (II) tris(bipyridine), also known as Ru(bpy)_3_^2+^, is a classic textbook example of an emissive TM complex which is widely reported as a photosensitizer. Lehn et al. first reported luminescence switching in TM complex dyad **C1** where the photoactive Ru(bpy)_3_^2+^ core was tethered to a quinone on one of the bipyridine ligands, which served as a quencher of excited triplet state Ru(bpy)_3_^2+^ [41]. Electrochemical reduction of quinone (in the presence of H^+^) to hydroquinone in **C1**, however, gave an λ_em_ of 608 nm. The fluorescence switching was reversible with electrical potentials between −0.6 to +1.1 V [41]. Years later, Zhong et al. developed a series of Ru(bpy)_3_^2+^ complexes, **C2a**–**c**, with multiple redox-active bis(4-methoxyphenyl)amino substituents decorated on the bipyridine ligands. Increase in the number of amine-functionalized bipyridine ligands from 1 to 2 and 3 decreased redox potentials and gave rise to λ_em_ of 655, 720, and 675 nm for **C2a**, **C2b**, and **C2c**, with ϕ_f_ of 3.0, 1.5, and 0.7%, respectively. Their emissions can be reversibly “switched off” by a one-electron-oxidation process whereby Ru^2+^ metal centres were oxidised to Ru^3+^ [42].

### 3.2. ELC Iridium Complexes

**Figure 10:**
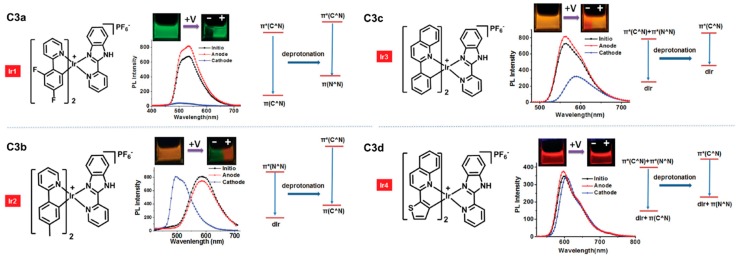
Photographs of ELC properties of **C3a**–**d** in acetonitrile and their emission spectra before and after application of 8 V. Also shown are the respective changes in ground and excited states’ energy levels. Reproduced with permission from [43]. Copyright 2016, Wiley-VCH Verlag GmbH and Co.

Huang et al. reported a series of ELC iridium(III) complexes in recent years. Firstly, they developed four ionic phosphorescent Ir(III) complexes, **C3a**–**d**, containing the same (2-(2-pyridyl)benzimidazole) ligand and different C^∧^N ligands, as shown in Figure 9 [43]. ELC properties were investigated by dipping two platinum electrodes into solutions of **C3a**–**d** in acetonitrile. As shown in Figure 10, the application of voltage (8 V) resulted in a change in emission colours of the solution around the negative electrodes with a clear boundary present. The colours of emission changes were associated with changes in HOMO-LUMO energy levels, which are dependent on the C^∧^N ligands coordinated: quenching of green emission for **C3a**, **C3b** from orange to green, and **C3c** from yellow to orange. Emission change was not obvious for **C3d**. By mixing the voltage-sensitive complexes with a voltage-insensitive luminophore as the background, multicolour tunable ELC devices were fabricated for information self-encryption and anti-counterfeiting applications [43].

**Figure 11:**
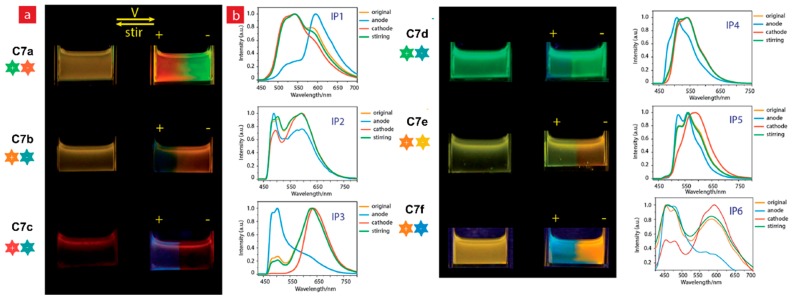
Photographs showing emission changes of ion pairs in acetonitrile upon application of 3 V voltage, with the corresponding luminescence spectra of the respective ion pairs shown on the right. Reproduced with permission from [45]. Copyright 2017, the Royal Society of Chemistry.

Later in the same year, the same group reported a series of six phosphorescent Ir(III) complexes, **4a**–**d** and **5a**–**b**, as well as two Ru(II) complexes, **6a**–**b**, containing pyrazine/pyrazinium- and pyridine/pyridinium-decorated ligands [44]. Pyrazine or pyridine-containing complexes **4b**, **4d**, **5b**, and **6b** exhibited from orange to NIR emission upon photoexcitation, whereas electron-deficient methylated pyrazinium or pyridinium-containing complexes **4a**, **4c**, **5a**, and **6a** were weakly or non- emissive, due to intramolecular photoinduced oxidative electron-transfer. When a voltage of 3 V was applied to solutions of the complexes in acetonitrile with n-butylammonium hexafluorophosphate, gradual photoluminescence turn-on was observed for **4a**, **4c**, **5a**, and **6a** at the cathode side, while the solutions around the anode remained non-emissive. This was attributed to the reduction of methylpyridinium and methylpyrazinium, which prevented oxidative quenching of excited complexes [44]. Complexes **4b**, **4d**, **5b**, and **6b**, however, did not show similar ELC behaviour. Subsequently, Huang et al. reported six luminescent ion pairs, **C7a**–**f**, made up of different emissive anionic and cationic Ir(III) complexes, to achieve a mixing of emission colours [45]. As shown in Figure 11, solutions of the six ion pairs in acetonitrile revealed yellow (**C7a**, **b**, **e**, **f**), red (**C7c**), and green (**C7d**) emission initially at the neutral state. Upon applying a voltage of 3 V, emission colour changes could be observed at both the positive and negative electrodes, attributing to ionic migration, and then reduction/oxidation occurring to individual cationic and anionic components of each ion pairs [45]. With stirring, the original emission states of the ion pairs could be recovered.

Zhao et al. recently studied the effects of counter anions on the emission colour tuning and switching properties of the Ir(III) complex containing 9-hexyl-3-pyridylcarbazole and 2,2′-dibenzimidazole ligands (**C8a**–**d**) [46]. Changing the counter-anion from Cl^−^ to Br^−^, then I^−^ and PF_6_^−^ produced red-shifting of emission with wavelengths from 493 to 590 nm and a corresponding change in emission colour from green to orange, due to the extent of anion-ligand interactions. Particularly, hydrogen bonding formed between N-H of 2,2′-dibenzimidazole ligands and F of PF_6_^−^ increases electron cloud density at the donor nitrogen atoms, thus leading to destabilization of the LUMO energy level, and giving rise to orange luminescence of **C8a**. The application of 5 V voltage in solution of **C8a** in acetonitrile led to the orange emission colour of solution around the cathode changing to green with a clear boundary observed. This is due to the ionic migration leading to non-homogenous distribution of cationic complex and PF_6_^−^ counter-anions over the two electrodes, thus, the extent of hydrogen bond formation between 2,2′-dibenzimidazole ligands and PF_6_^−^ which was translated to the charge cloud polarization of the ligand [46].

## 4. ELC Polymers

Compared to small molecules and metal complexes, polymers offer the advantage of better processability and, in particular, better film homogeneity, which are very much desired for device fabrication. As such, there are considerably more examples of ELC polymers compared to that of small molecules. Most of these reported ELC polymers are fluorescent in the neutral state, and the fluorescence intensity can be modulated by an external electric bias. This review categorises the ELC polymers into two classes: non-conjugated and conjugated polymers. In the former class, electroactive fluorophore moieties are connected to each other in the polymer backbone via non-conjugated structural linkages or groups and, hence, there is lesser electronic communication between them. This brings about the advantage of improved physical properties, structural stability, as well as better processability, compared to individual fluorophores in a small molecule form. In the latter class, the entire polymer chain is conjugated and, hence, efficient electronic communication with an extensive charge flow across the entire polymer chain exists. This, however, allows scientists to design and synthesize conjugated polymers with tunable bandgaps and, thus, enables control of the physical and emission colours.

### 4.1. Non-Conjugated Polymers

**Figure 12:**
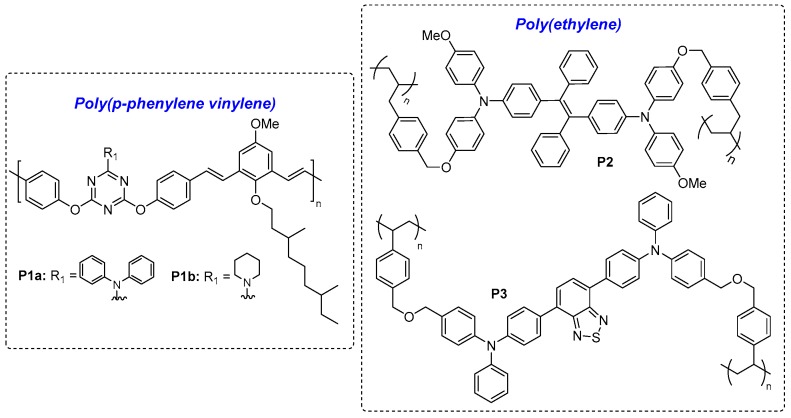
Chemical structures of ELC poly(p-phenylene vinylene) and poly(ethylenes).

**Figure 13:**
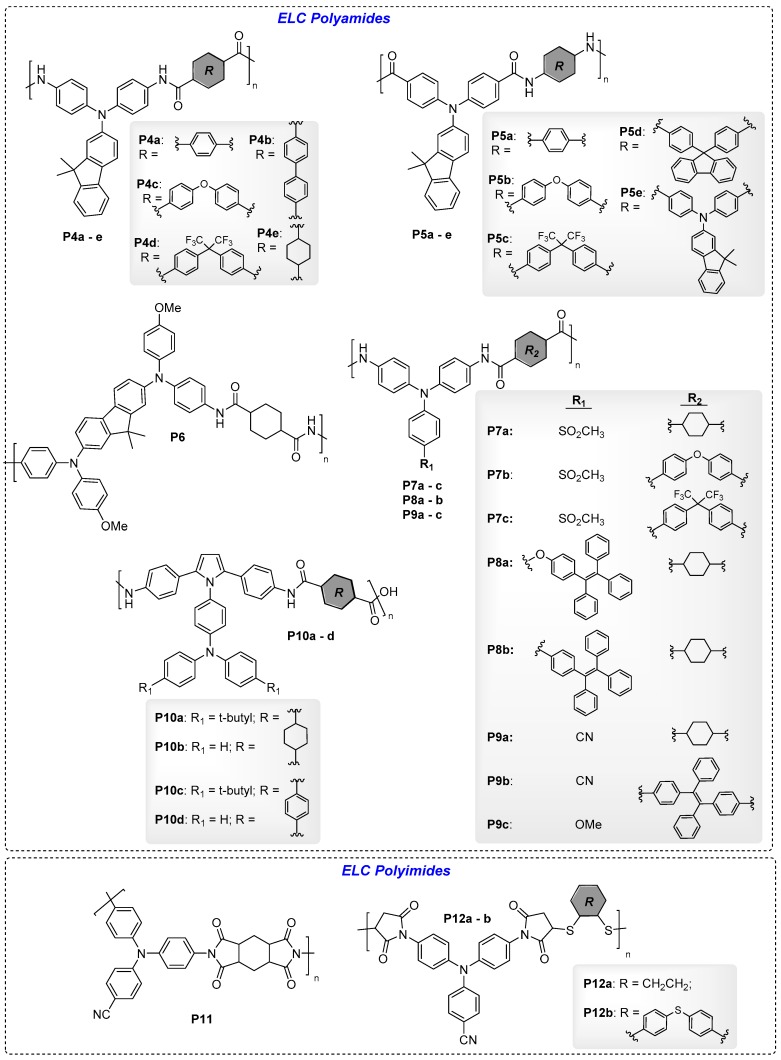
Chemical structures of ELC polyamides and polyimides.

Figure 12, Figure 13, and Figure 16 show the chemical structures of ELC non-conjugated polymers. In many examples, electroactive triarylamine moieties are frequently used to generate luminescence as well as ELC properties. They are often connected to each other in the polymer backbone via ether linkages or in the forms of polyimides and polyamides. The ELC parameters of these examples are summarised in Table 4.

#### 4.1.1. ELC Poly(p-Phenylene Vinylene) and Poly(Ethylenes)

One of the earliest examples of ELC non-conjugated polymers reported by Kim et al. was *p*-phenylene vinyl polymer containing a redox-active s-triazine spacer, **P1a**–**b** [47]. Fluorescent monomers with two peripheral styryl moieties allow opportunities for the preparation of cross-linking poly(ethylenes). Joseph et al. reported cross-linked polymer **P2** with aggregation-induced emission (AIE) tetraphenylethylene (TPE) units, giving a highly emissive thin film device with reversible fluorescence quenching [48]. Similarly, Skene et al. also reported cross-linked polymer **P3** containing a benzothiadiazole acceptor flanked by two triphenylamine units, which caused red-shifting of λ_em_ to 630 nm in thin film [49]. The fluorescence intensity can be switched by successive oxidation and reduction back to the neutral state, though the latter only restored 50% of its original fluorescence.

#### 4.1.2. ELC Poly(amides) and Poly(imides)

**Figure 14:**
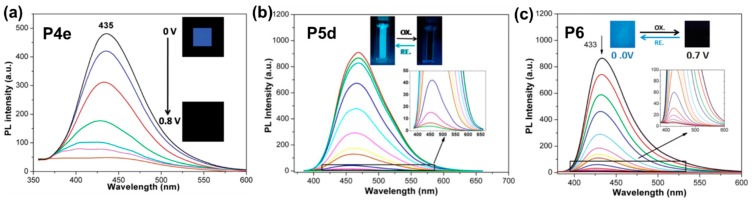
Photoluminescence (PL) spectra changes of polymers **P4e** (**a**), **P5d** (**b**), and **P6** (**c**) thin film devices with increasing applied potentials. Inserts are photos showing the fluorescence changes of the three polymers thin films. Reproduced with permission from [50] (**a**), [51] (**b**), and [52] (**c**). Copyright 2015 and 2016, the Royal Society of Chemistry; and 2016 Wiley Periodicals, Inc.

Fluorene is a well-known highly-fluorescent compound that is widely used to develop optoelectronics materials. Chen et al. reported a series of non-conjugated ELC polyamides containing electroactive triarylamine moieties: **P4a**–**e**, with *N,N*-diphenyl-fluoren-2-amine attached to the nitrogen (NH) side of amide linkages, [50] and **P5a**–**e**, with *N,N*-diphenyl-fluoren-2-amine attached to the carbonyl side (C=O) side of amide linkages [51]. A series of connecting spacers were used in the polyamides: phenyl, diphenyl, oxydiphenyl, (perfluoropropane-2,2-diyl)diphenyl, and cyclohexyl, etc. Amongst the first series of polyamides, **P4e** with cyclohexyl spacer exhibited a significantly high ϕ_f_ of 47.1% in solution (vs. 0.37–0.68% of **P4a**–**d**) due to a locally excited HOMO-LUMO transition over the fluorene-based amine and the absence of inter-chain charge transfer experienced in **P4a**–**d**. Thin film device of **P4e** exhibited reversible quenching of blue fluorescence as the applied voltage increased from 0 to 0.8 V with an I_OFF/ON_ of 12.7 due to the formation of triarylamine radical mono-cations (Figure 14a) [50]. For the second series of polyamides, λ_em_ was generally blue-shifted compared to that of the first series (448–492 nm vs. 441–581 nm) with **P5b**–**d** having significantly higher ϕ_f_ compared to **P5a** and **P5e** (25.7–34.1% vs. 2.0 and 9.2%). In particular, the thin film EC device of **P5d** exhibited blue-green fluorescence, which was quenched to dark on increasing the applied potential from 0 to 1.3 V, due to the formation of the triarylamine radical cation (Figure 14b). Compared to **P4e**, fluorescence switching of **P5d** revealed a remarkably high contrast ratio of 221.4 [51]. Additionally, incorporating highly-fluorescent fluorene moiety as a building block, Chen’s group also reported highly fluorescent polyamide **P6** with bis(diphenylamino)fluorene as the electroactive fluorophore, an aliphatic cyclohexyl spacer which was established to reduce the inter-chain charge transfer effect, hence, leading to stronger fluorescence [52]. The polymer film emitted blue fluorescence which reversibly quenched with an optical contrast of 152 upon oxidation (Figure 14c).

Electroactive triphenylamine is a key building block for EC materials due to its ability to form stable radical cations at the nitrogen atom upon oxidation [30]. Three polyamides, **P7a**–**c**, containing sulfone groups substituted on triphenylamine were reported [53]. Amongst the three polyamides, **P7a** reveal aggregation-enhanced emission (AEE) properties with a high solid state ϕ_f_ of 32.1% and a remarkably high switching contrast of 234. TPE is a well-known AIE fluorophore and is extensively used in the structural designs of a wide range of materials for optoelectronics, chemosensing, and bioimaging applications [8,9,10,11]. The AIE properties is particularly useful in materials for optoelectronic (displays and lighting) devices, including ELC devices, so as to achieve bright photoluminescence in the solid thin film state and hence provide high contrast ratios. Two AIE-active polyamides, **P8a** and **b**, containing AIE-active TPE group attached to the electroactive triphenylamine moiety via an ether linkage, and TPE directly attached to the *N*- position of diphenylamine, respectively, were reported [54,55]. Both AIE-active polymers produced highly emissive thin films, which exhibited fluorescence switching with very high optical contrasts of 206 and 417, respectively.

**Figure 15:**
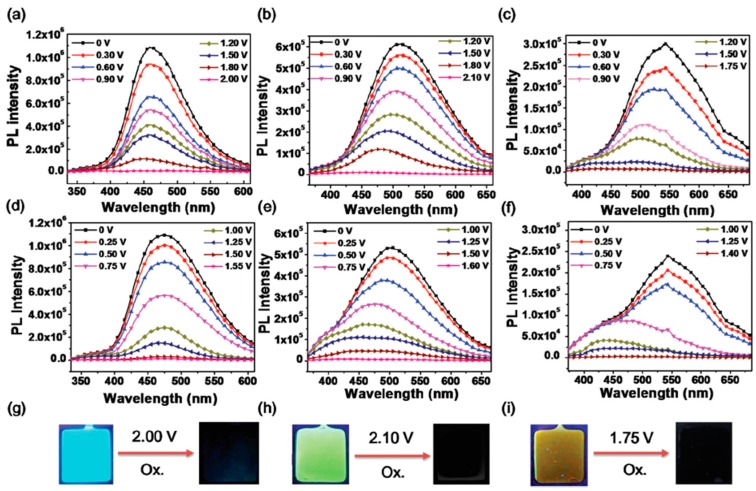
Photoluminescence spectra of **P9a** (**a**), **P9b** (**b**), and **P9c** (**c**) thin film devices without HV at different applied voltages. The photoluminescence spectra of **P9a**, **P9b**, and **P9c** thin film devices with HV are as shown in (**d**–**f**), respectively. Photos showing the corresponding fluorescence changes of **P9a** (**g**), **P9b** (**h**), and **P9c** (**i**) on oxidation are as shown. Reproduced with permission from [56]. Copyright 2018, the Royal Society of Chemistry.

Liou’s group also developed a series of EFC polyamides, **P9a**–**c**, bearing triphenylamine in the backbone and studied the effects of different substituents on the triphenylamine moieties (electron-withdrawing cyano group and electron-donating methoxy group), as well as the effects of different spacers (cyclohexyl and TPE) in the polymer chain [56]. Interestingly, **P9b** and **P9c** containing AIE-active TPE moieties exhibited a lower solid state ϕ_f_ than cyclohexyl-containing **P9a**, which was due to the TPE moiety being a stronger electron-donor than cyclohexane, thus contributing to a greater overall PET effect. Electrochemical oxidation of the three polyamide films quenched their fluorescence as shown in Figure 15, with a response time of 8.6, 7.1, and 6.5 s, respectively [56]. Furthermore, by incorporating hexyl viologen (HV) into the thin film EFC devices, fluorescence switching contrast ratio of **P9a**, **P9b**, and **P9c** could be increased from 21, 13, and 10% to 51, 43, and 37%, respectively.

EC-active triphenylamine groups could be attached to the polymeric backbone as side chains in the design of novel EC and EFC polymers. For example, Wang and Niu et al. reported a series of four polyamides **P10a**–**d** containing an electroactive fluorophore with 2,5-diphenylpyrrole attached to triphenylamine at the pyrroles’ *N*- position [57]. Amongst these, **P10a** and **b** exhibited violet fluorescence in the neutral state which was gradually weakened with increasing positive potentials due to the formation of the fluorescence-quenching radical cation over the electroactive fluorophore.

Several EFC polyimides were also reported by Liou’s group. Firstly, AEE-active polyimide **P11** bearing cyano-substituted triphenylamine was reported [58]. EFC thin film devices of **P11** emitted blue fluorescence which was quenched as applied potentials increased from 0 to 1.6 V, with a remarkable I_OFF/ON_ contrast ratio of 151.9. More recently, Liou et al. reported two novel cyanotriphenylamine-bearing polyimidothioethers **P12a** and **P12b** [59]. Thin films of **P12a** and **P12b** possessed a λ_em_ at 427 and 429 nm, with a photoluminescence ϕ_f_ of 10.4% and 6.1%, respectively. The fluorescence was quenched upon the application of positive voltage from 0 to 1.7 V due to the formation of the TPA radical cation. On the other hand, applying a negative potential up to −2.0 V also led to fluorescence quenching due to reduction of imide’s carbonyl groups into the radical anion state. A contrast ratio of up to 92 was registered for the reversible fluorescence switching process [59].

#### 4.1.3. ELC Block Co-Polymers

**Figure 16:**
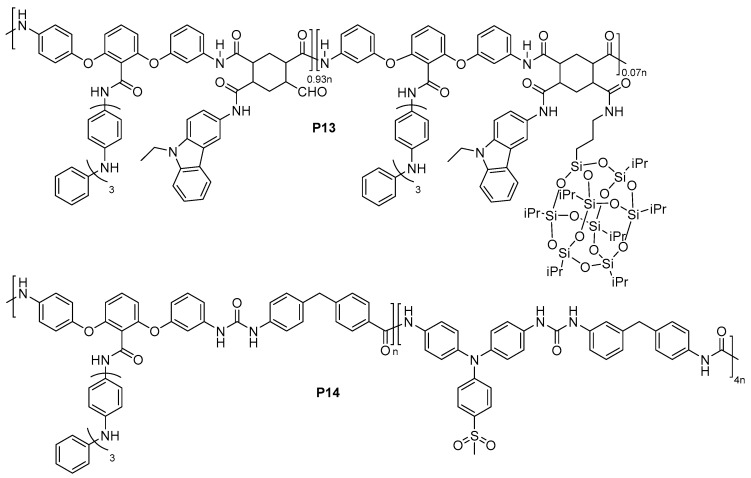
Chemical structures of EFC block co-polymers **P13** and **P14**.

**Figure 17:**
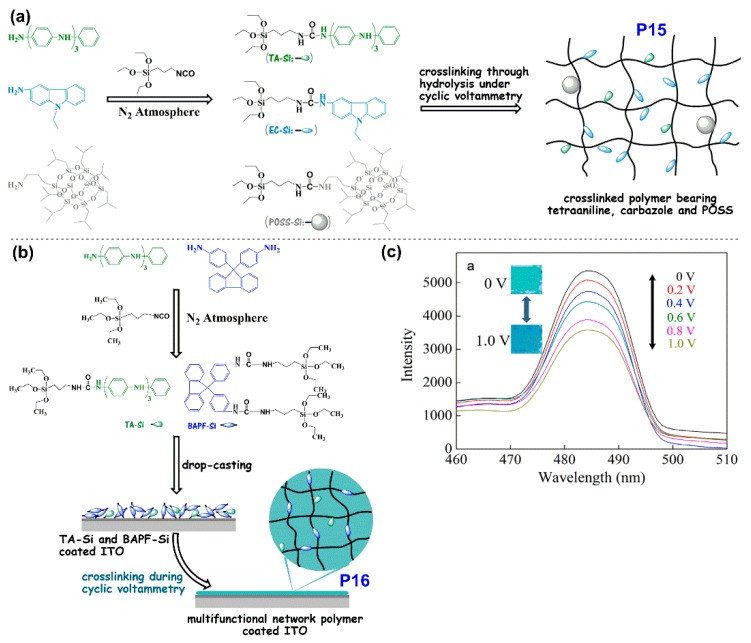
Schematic diagram of preparation of cross-linked polymers **P15** (**a**) and **P16** (**b**). The fluorescence spectra of **P16** at different applied positive potentials with photos of fluorescence change is as shown in (**c**). Reproduced with permission from [61,62]. Copyright 2018, Elsevier B. V.

In recent years, Chao’s group developed a series of random block co-polymers and cross-linked EFC polymers (Figure 16 and Figure 17). Block co-polymer **P13** was developed, containing oligoaniline groups as the electroactive unit and carbazole group as the fluorescence emission unit, both attached to the main polymer chain as pendants via amide linkage [60]. In addition, the bulky polyhedral oligomeric silsequioxane (POSS) group was also included as a pendant group to create steric hindrance and, hence, mitigate the aggregation-caused quenching effect of the fluorescence. Oxidation of **P13** generates quinoidal oligoaniline which acts as excitation traps and reversibly quenches the blue fluorescence of carbazole with a contrast of up to 85% and a quick switching time of 11.2 s [60]. Chao et al. similarly utilized sterically-bulky POSS groups in the development of a cross-linked polymer network **P15** consisting of tetraaniline, carbazole, and POSS, which was synthesised via hydrolysis reaction during cyclic voltammetry, as shown in Figure 17a [61]. Following the same quinoidal-mediated fluorescence-quenching mechanism as **P13**, blue emission (λ_em_ = 486 nm) of the drop-casted polymer thin film was quenched by 14% as an applied potential increased from 0 to 1.0 V with switching times of 10.5 s/9.2 s for the on/off states [61]. Another crosslinked polymer **P16** with oligoaniline and fluorene groups were synthesised via a hydrolysis reaction during cyclic voltammetry of drop-casted monomers, as shown in Figure 17b [62]. Similarly, fluorescence of **P16**, which originates from the fluorene moieties, was partially quenched with increase in applied positive potentials (Figure 10c), registering a contrast of up to 60% and off/on switching times of 2.4/4.2 s, respectively. By introducing two electroactive groups, oligoaniline and methylsulfonyl triphenylamine, into the same block co-polymer, polyuria **P14** exhibited multistage regulated EC property with a transmittance optical contrast of 38% [63]. The fluorescence of **P14** originates from methylsulfonyl triphenylamine and its switching was found to depend on the redox transition of the oligoaniline pendants component. Similar to the above two examples, oxidation of **P14** results in the formation of quinoidal oligoaniline, serving as excitation traps which quenches fluorescence (λ_max_ = 518 nm). The fluorescence off and on switching time was recorded to be 9.4 s and 10.8 s, respectively [63].

### 4.2. Conjugated Polymers

**Figure 18:**
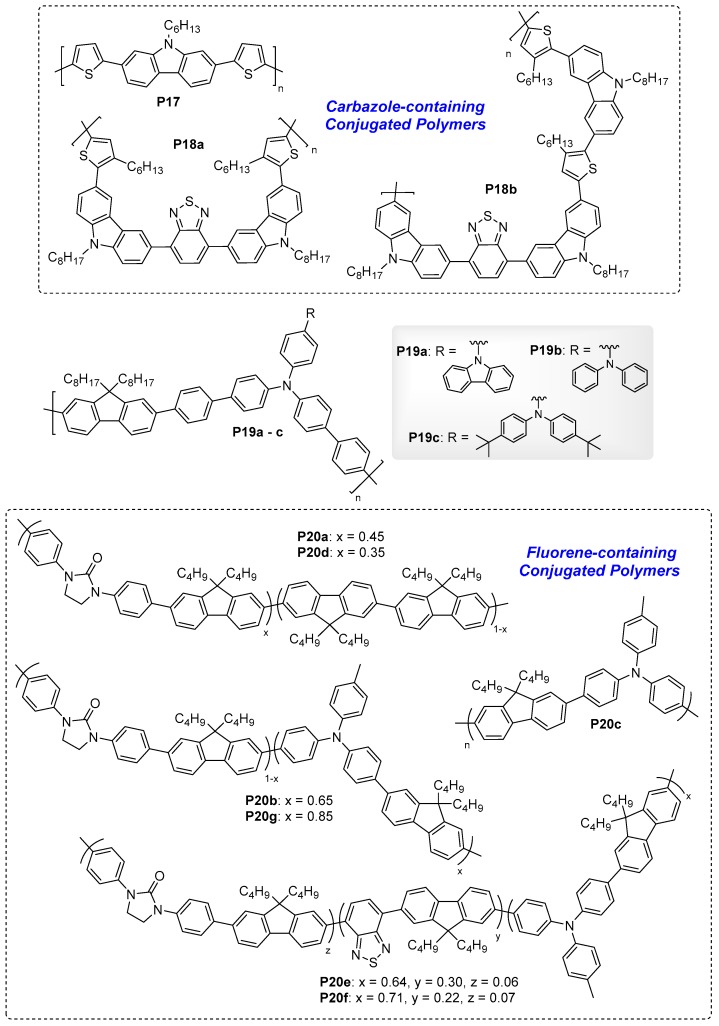
Chemical structures of ELC conjugated polymers containing carbazole and fluorene.

**Figure 19:**
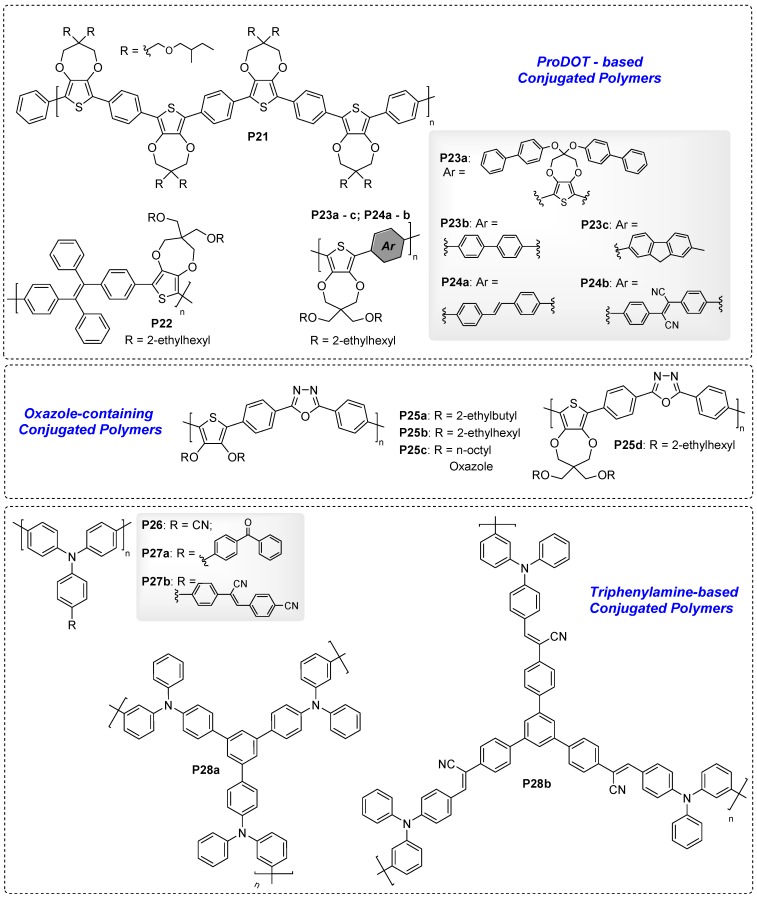
Chemical structures of ELC-conjugated polymers containing ProDOT, oxazole, and triphenylamine.

Unlike non-conjugated polymers, the efficient charge communication and electron flow can prevail across the entire chain of conjugated polymers. As such, fluorescent conjugated polymers offer the opportunity to tune physical and emission colours via molecular engineering, i.e., the use of different donors and acceptors moieties. Figure 18 and Figure 19 show the chemical structures of key EFC fluorescent conjugated polymers with their key ELC parameters (Table 5). In this section, four types of EFC conjugated polymers including carbazole-, fluorine-, propylenedioxythiophene-, and oxazole-containing conjugated polymers are summarised.

#### 4.2.1. ELC Carbazole-Containing Conjugated Polymers

Carbazole is a useful redox-active building block used for the development of EC materials [64,65,66]. Goto et al. reported dithienyl-carbazole-based conjugated polymer **P17** which exhibited both EC and ELC properties [67]. Thin films of **P17** appeared yellow with light-green emission upon irradiation under a UV lamp. Electrochemical oxidation, however, changed the film colour to black and quenched the fluorescence due to the formation of radical cations (polarons) and dications (bipolarons) along the conjugated polymer backbone [67]. Xu et al. separately reported two carbazole-benzothiadiazole co-polymers **P18a** and **18b** with both EC and EFC properties that were found useful for the detection of cyanide anions [68,69]. Nanoporous copolymer **P18a** exhibited an oxidative quenching with a high contrast ratio of 19.2 in the absence of cyanide anions [69]. Similarly, a thin film device of **P18b** spin-coated on ITO/poly(ethylene terephthalate) experienced up to 80% quenching of fluorescence upon undergoing electrochemical oxidation, due to the formation of carbazole radical cation and dications [68]. Their oxidative quenching effect was, however, weakened in the presence of cyanide due to the interaction between the cyanide nucleophile and electron-deficient benzothiadiazole, showing potential to detect cyanide anions.

#### 4.2.2. ELC Fluorene-Containing Conjugated Polymers

While we have discussed fluorene-containing ELC polyamides in the previous section, the highly fluorescent fluorene moiety is also used in the development of ELC-conjugated polymers. Wang et al. recently reported a series of conjugated polymers **P19a**–**c** containing fluorene and triphenylamine moieties [70]. This series of polymers **P19a**–**c** revealed blue-to-dark emission change as applied external potentials increased from 0 to 1.5 V. As anticipated, the fluorescence-quenching of both series of polymers was due to the formation of the triphenylamine radical cation [70].

**Figure 20:**
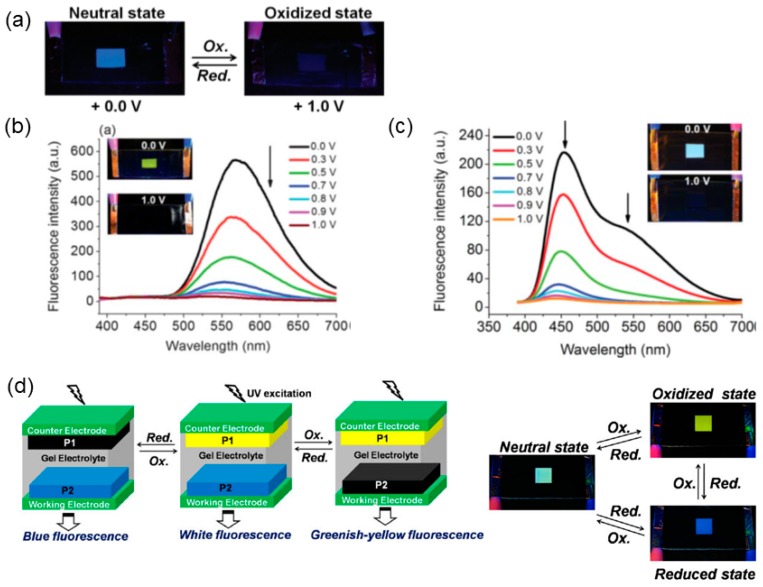
(**a**) Photographs showing blue-fluorescence-switching of **P20b** between neutral and oxidised states. Reproduced with permission from [71]. Copyright 2012, Wiley Periodicals, Inc. (**b**) Fluorescence intensity spectra of **P20e** at different applied positive potentials, with photographs showing the corresponding yellow-fluorescence-switching. (**c**) Fluorescence intensity spectra of the EFC device comprising a blend of **P20b** and **P20e** at different applied positive potentials, with photographs showing the corresponding white-fluorescence switching. Both (**b**,**c**) reproduced with permission from [72]. Copyright 2013, the Royal Society of Chemistry. (**d**) Schematic illustration of multicolour EFC device comprising **P20e** and **P20f**, with photographs showing the fluorescence switching between blue, white, and greenish-yellow. Reproduced with permission from [73]. Copyright 2014, the American Chemical Society.

Leung et al. successively reported a series of conjugated co-polymers **P20a**–**b, d**–**f** containing highly-fluorescent fluorene moiety, and a dipolar cyclic urea group, imidazolidin-2-one. **P20a** and **P20b** were synthesised by co-polymerizing the monomer containing 1,3-diphenylimidazolidin-2-one and 9,9′-dibutylfluorenyl, with 2,2′-bifluorenene monomer and fluoren-2-yl-(4-methyltriphenylamine) monomer, respectively [71]. Polymer **P20c** containing only 4-methyltriphenylamine and 9,9′-dibutylfluorene was also synthesised for comparative study purpose. Thin films of **P20a** and **P20b** spin-coated onto ITO-coated glass substrate exhibited bright blue fluorescence with emission λ_max_ at 423 and 443 nm, and high quantum efficiencies of 77% and 73%, respectively. Gradual weakening and subsequent quenching of fluorescence were observed for **P20b** upon electrochemical oxidation, with a contrast ratio of 16.3, as shown in Figure 20a. On the other hand, **P20a** only experienced partial quenching of fluorescence due to the absence of electroactive triphenylamine moiety (present in **P20b**) that forms a fluorescence-quenching radical cation upon undergoing oxidation. Leung et al. then reported co-polymers **P20d** and **P20e** containing additional benzothiadiazole moiety that red-shifted absorption and emission wavelengths for **P20d** and **P20e** [72]. The two polymers emitted greenish-yellow and yellow fluorescence in solid thin film under UV light, with emission λ_max_ of 556 and 565 nm, but with significantly lower quantum efficiencies of 23% and 17%, respectively. Similarly, both exhibited reversible fluorescence quenching upon undergoing electrochemical oxidation, with a contrast ratio of 21.4 recorded for **P18e** at a switching pulse time of 10 s (Figure 20b). Blending **P20e** and **P20b** together in a 2:1 ratio, a white-light EFC switching device could be obtained with reversible fluorescence switching and a contrast ratio up to 14.6 s (Figure 20c) [72]. Finally, blue-emitting EFC polymer **P20f** was synthesised and incorporated into an EFC device together with greenish-yellow-emitting **P20e**, but coated onto two independent ITO electrodes, effectively giving rise to a single device with electrically-switchable multi-fluorescence states [73]. Figure 20d shows the multicolour EFC device structure and how fluorescence of the dual-polymer device could be reversibly switched between blue (at negative bias), greenish-yellow (at positive bias), and white (at neutral bias) by successively quenching the fluorescence of each polymer.

#### 4.2.3. ProDOT-Based ELC-Conjugated Polymers

Kim et al. also reported phenyl-propylenedioxythiophene (ProDOT)-based conjugated polymer **P21** which quantum efficiency changed from 3.8 to 0.21% during fluorescence switching. In addition, with ProDOT units bearing chiral alkyl substituents, **P21** showed a negative bisignate Cotton effect, with a chemically-switchable chirality of 97% change in the anisotropy factor [74].

**Figure 21:**
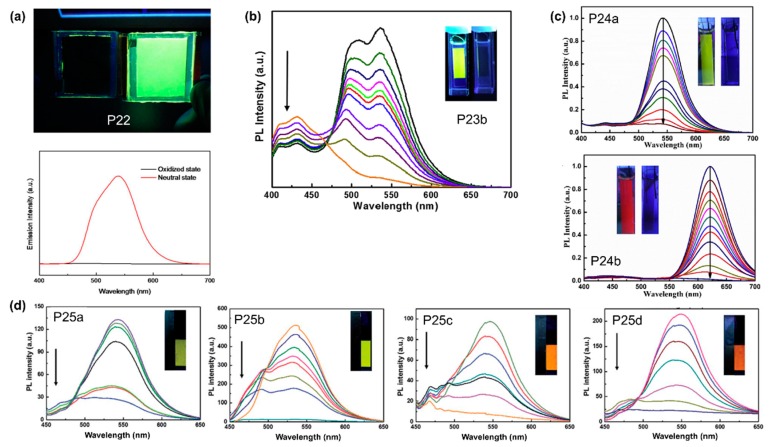
Photographs and photoluminescence spectra of EC/EFC devices containing **P22** (**a**); **P23b** (**b**); **P24a** and **P24b** (**c**); and **P25a**–**d** (**d**) showing photoluminescence quenching upon undergoing electrochemical oxidation from the neutral to oxidised states. Reproduced with permission from [75] (**a**), [76] (**b**), [77] (**c**) and [78] (**d**). Copyright 2015, the American Chemical Society (**a**); 2017 and 2018, Elsevier B. V. (**b**,**c**); and 2017, the Royal Society of Chemistry.

In recent years, Xu’s group developed a series of conjugated polymers containing ProDOT moieties that were found to be bifunctional, i.e., that exhibit both EC and EFC properties. To begin with, Xu et al. copolymerised ProDOT with AIE-active TPE units to afford polymer **P22** [75]. Solid thin films of **P22** emit strong yellow fluorescence due to AIE properties induced by TPE, and upon electrochemical oxidation, this fluorescence was quenched, as shown in Figure 21a, due to oxidation selectively occurring at TPE moieties to become radical cations [75]. Xu et al. then reported a series of polymers **P23a**, **P23b**, **P23c**, **P24a**, and **P24b** in which ProDOT was copolymerised with 1,1′-biphenyl-4-oxy-functionalized ProDOT, 1,1′-biphenyl, fluorene, stilbene, and 1,2-diphenylfumaronitrile, respectively [76,77]. For the first series of polymers, **P23a**–**P23c**, only **P23b** with biphenyl groups exhibit solid state fluorescence due to AIE effect resulted from intramolecular rotation and conformational twisting. Upon increasing an applied positive potential from −1 to 1.6 V, bright yellow fluorescence of **P23b** gradually faded to darkness (Figure 21b) with a transmittance change of 35%, possibly due to the formation of radical cations [76]. Incorporating stilbene and 1,2-diphenylfumaronitrile co-monomers into **P24a** and **P24b**, respectively, leads to forming C–H^…^π interactions between neighbouring polymer chains, making it useful in avoiding undesirable exciton interactions in the aggregation state. Like **P23**, thin film of **P24a** exhibited yellow-green emission which was quenched upon electrochemical oxidation (Figure 21c). The emission of **P24b**, however, was red-shifted to λ_max_ of 612 nm due to the presence of cyano groups. The red fluorescence of **P24b** thin film was quenched with increasing positive potentials applied, presumably due to the formation of radical cations as well (Figure 21c) [77].

#### 4.2.4. ELC-Conjugated Polymers Containing Oxazole

In this section, a series of bifunctional polymers, **P25a**–**d**, copolymerised between 2,5-diphenyl-1,3,4-oxadiazole and 3,4-dialkyoxythiophenes (DalkOTs) or ProDOT were synthesized by Xu’s group in an effort to combine fluorescence properties of oxadiazole and EC properties of thiophene derivatives [78]. The side chains on DalkOT moieties of **P25a**–**c** varied to adjust the extent of steric hindrance and, hence, potentially control polymer colour and fluorescence properties. Changing the applied potential of individual thin film devices from −1.6V to 1.8 V, the bright yellow fluorescence of **P25a**–**b** and orange fluorescence of **P25c**–**d**, were effectively quenched, as shown in Figure 21d, due to the formation of radical cations [78].

#### 4.2.5. Triphenylamine-Based ELC-Conjugated Polymers

Triphenylamine is a classical electroactive building block for both EC and ELC materials. Liou et al. reported polymer **P26** prepared by FeCl_3_-mediated oxidative coupling polymerization of cyanated triphenylamine monomers [79]. **P26** thin film revealed bright bluish-green fluorescence with quantum efficiency of 21.9%. Upon applying external voltage from 0 to 1.9 V, quenching of fluorescence occurs due to the formation of triphenylamine radical cation with a remarkable contrast ratio (I_OFF/ON_) of 242, a rapid response time of <0.4 s and outstanding device stability of 99% fluorescence recovery after 300 cycles [79]. Liang et al. reported two triphenylamine-based homopolymers, **P27a** and **P27b**, which were functionalized with benzophenone and 7-cyano-(E)-stilbene on the third phenyl ring of triphenylamine monomer, respectively [80]. The electron-withdrawing substituents were supposed to impose intramolecular charge transfer (ICT) effect, which appeared more prominent for **P27b**, as evident of its higher electrochemical oxidation potential and lower ion diffusion coefficient. Both polymers exhibited multichromic EC properties. Using **P27b** as an example, it showed the change from yellow to orange, then brown and finally dark grey on electrochemical oxidation. Green fluorescence of both polymer films was quenched upon oxidation as well [80]. More recently, Malik et al. reported triphenylamine-based dendritic macromolecules **P28a** and **b** which were synthesised via electrochemical polymerization. The emission of **P28a** and **b** changed from blue to dark, and yellow to dark, respectively, upon oxidation, with contrast ratios of 64 and 179, respectively [81].

## 5. Conclusions and Perspective

In summary, this review has reviewed ELC materials from various small organic molecules, transition metal complexes, and polymers. In small organic molecules, by tethering a non-electroactive fluorophore to a redox-active moiety in the form of a dyad, or in intrinsically electroactive fluorophores such as those viologens or arylamine-containing molecules, electroluminochromism could be achieved. Compared to small molecules, polymers offer additional advantages, such as mechanical robustness, physical stability, and excellent processability, and are gradually attracting more attention in the field of ELC materials. Electroactive fluorophores, such as arylamines and carbazoles, could be connected along the polymeric backbone or attached as sidechains as non-conjugating groups, in the forms of polyamides or polyimides. Alternatively, they could be coupled to each other or with other conjugated units, either acceptors or donors, in the form of conjugated polymers, which offer the opportunity to tune the optical bandgap and, hence, modulate their emission and physical colours via molecular engineering. Luminescent transition metals complexes, particularly those with Ru(II) and Ir(III) centres could also serve as good candidates for ELC materials with reversible phosphorescence switching. Nonetheless, ELC materials are not restricted to these three broad categories as there were also other types of compounds, such as polyoxometalates (POM) and quantum dots (QD), which have been used for the development of ELC mixed-systems [82,83,84,85,86,87,88]. Due to the scope of this review, these examples will not be discussed in detail in this paper.

ELC materials are widely applicable in optoelectronics and sensing applications. However, research in the development of ELC materials is still lacking compared to EC materials. Optimistically, this means that there is still much room for us to explore novel ELC materials and translate these materials into useful applications. Several aspects of ELC performance currently reported could be further improved. For instance, most of the ELC organic molecules and polymers cited in this paper exhibited fluorescence quenching upon electrochemical oxidation. As such, perhaps research efforts could be invested into the design and synthesis of novel organic materials with multi-luminochromic states, i.e., switching between two or more different emission colours (instead of quenching only) via control of the external electrical voltage applied. The multi-luminochromic phenomenon was prevalent in some examples of the TM complexes, but it was rarely seen in organic ELC material systems. Equally importantly, research could also be channelled into device fabrication and optimization, aiming to develop prototypes for advanced applications, such as printable optoelectronics or wearable devices, towards eventual commercialization of ELC technologies, and there is no doubt that there are still many possibilities for the future of ELC material development.

## Figures and Tables

**Table 1:** ELC parameters of EFC molecular dyads.

	Neutral State				Oxidised State		Ref.
	λ_abs_ (nm)	λ_em_ (nm)	Φ	Emission Colour	Changes/Effects on Emission	Contrast Ratio I_OFF/ON_	
**M1**	382	385, 405, 425	0.0035	Blue	Enhanced; Φ = 0.059	1.3	[16]
**M2**	ca. 490, 525	533, 577, 625	0.0039	Red	Enhanced	-	[17]
**M3**	342, 590, 739	-	-	-	Emission Turn-on at λ = 610 nm	-	[18]
**M4**	334, 518	385, 558	0.32	Yellow	Quenched	-	[20]
**M5a**	255, 342, 520	418	0.004	Blue	Quenched	-	[21]
**M5b**	257, 326, 511	415	0.001	Blue	Emission Turn on at λ = 550 nm	-	[21]
**M5c**	258, 337, 511	417	0.008	Blue	Emission Turn on at λ = 558 nm	-	[21]
**M5d**	345, 507	426	0.06	Blue	-	-	[21]
**M6**	754	803	0.43	-	Emission quenched, then turn on at λ = 1185 nm (Φ = 0.00048)	-	[22]

**Table 2:** ELC performance parameters of electroactive and redox-active fluorophores.

	Neutral State				Oxidised State			Ref.
	λ_abs_ (nm)	λ_em_ (nm)	Φ	Emission Colour	Changes/Effects on Emission	Contrast Ratio I_OFF/ON_	Response Time, *t* (s)	
**M7**	795	822	-	NIR	Quenched	1.5	-	[26]
**M8a**	675	711	0.13	NIR	Quenched	86.4	-	[27]
**M8b**	694	741	0.36	NIR	Quenched	76.3	-	[27]
**M8c**	762	803	0.01	NIR	Partially Quenched	40.2	-	[27]
**M9a**	687	721	0.28	NIR	Partially Quenched	25.5	-	[27]
**M9b**	696	748	0.24	NIR	Partially Quenched	55.2	-	[27]
**M10a**	566, 562	593	1.00	-	Quenched	-	-	[28]
**M10b**	580, 582	614	0.89	-	Quenched	-	-	[28]
**M10c**	578	600	0.76	-	Quenched	-	-	[28]
**M11a**	470	680	0.04	Purple	Emission colour changes from purple to blue	-	-	[29]
**M11b**	500	702	<0.01	Grey-green	Emission colour changes from grey-green to green	-	-	[29]
**M12a**	353	531	0.06	Green	Quenched	-	-	[31]
**M12b**	355	532	0.04	Green	Quenched	-	-	[31]
**M12c**	311	447	0.09	Blue	Quenched	-	-	[31]
**M12d**	283	384	0.06	Blue	Partially Quenched	-	-	[31]
**M12e**	301	382	0.03	Blue	Partially Quenched	-	-	[31]
**M12f**	357	418	0.37	Blue	Quenched	-	-	[31]
**M13**	346	424	0.39	Blue	Stepwise Quenching (Off-On-Off)	5	-	[32]
**M14a**	415	474, 496	0.71	-	Emergence of electro-chemiluminescent	-	-	[33]
**M14b**	447	546	0.67	-	Emergence of electro-chemiluminescent	-	-	[33]
**M15**	360	388	0.40	-	Emergence of electro-chemiluminescent	-	-	[33]
**M16**	Ca. 420	446	0.30	Blue	Stepwise Quenching	-	-	[34]
**M17a**	432 ^	640 ^	0.03 ^	Red	Quenched	-	-	[35]
**M17b**	432 ^	640 ^	0.075 ^	Red	Quenched	-	-	[35]
**M18a**	430	530	0.673	Green	Quenched	66–79	3.1 (on) 24.2 (off)	[36]
**M18b**	430	530	0.60	Green	Change to red	76.4	3.6 (on) 3.5 (off)	[36]
**M19a**	390	Ca. 455	0.92	Blue	Quenched	-	-	[38]
**M19b**	390	Ca. 455	0.87	Blue	Quenched	-	-	[38]
**M19c**	395	461	0.79	Blue	Quenched	-	-	[38]
**M20a**	510	429	-	Blue	Partially Quenched	-	9.5	[39]
**M20b**	530	463	-	Brilliant Blue	Partially Quenched	-	18.4	[39]
**M20d**	592	547	-	Yellow	Partially Quenched	-	24.0	[39]

^ Denotes optical properties of aggregates measured in THF/water 1:9 *v/v*.

**Table 3:** ELC parameters of transition metal complexes.

	Neutral State				Oxidised State		Ref.
	λ_abs_ (nm)	λ_em_ (nm)	Φ	Emission Colour	Changes/Effects on Emission	Contrast Ratio I_OFF/ON_	
**C1**	453	610	-	Deep Red	Enhances on Reduction ^	-	[41]
**C2a**	478	655	0.03	Deep Red	Quenched	-	[42]
**C2b**	505	720	0.015	NIR	Quenched	-	[42]
**C2c**	528	675	0.007	NIR	Quenched	-	[42]
**C3a**	-	Ca. 540	-	Green	Quenched on Reduction	-	[43]
**C3b**	-	Ca. 590	-	Orange	Green on Reduction	-	[43]
**C3c**	-	Ca. 560	-	Yellow	Orange on Reduction	-	[43]
**C3d**	-	Ca. 600	-	Red	No Change	-	[43]
**C4a**	Ca. 290, 330, 425	n.d.	<0.001	-	-	-	[44]
**C4b**	Ca. 290, 325, 425	720	0.018	-	-	-	[44]
**C4c**	Ca. 260, 325, 425	580	<0.001	Non Emissive	Turn on Red PL at cathode	26	[44]
**C4d**	Ca. 360	640	0.036	-	-	-	[44]
**C5a**	Ca. 260, 400	635	<0.001	-	Turn on Red PL at cathode	-	[44]
**C5b**	Ca. 260, 375	650	0.019	-	-	-	[44]
**C6a**	Ca. 250, 295, 360, 470	725	0.005	-	Blue-shift and turn on red PL at cathode	-	[44]
**C6b**	Ca. 250, 290, 325, 430, 500	655	0.075	-	-	-	[44]
**C7a**	-	550, 600	0.08	Yellow	Red at anode, green at cathode	-	[45]
**C7b**	-	472, 510, 580	0.35	Yellow	Teal at anode, Orange at cathode	-	[45]
**C7c**	-	472, 510, 630	0.13	Red	Blue at anode, Red at Cathode	-	[45]
**C7d**	-	472, 510, 550	0.37	Green	Darker green at anode, Green at cathode	-	[45]
**C7e**	-	520, 580	0.20	Yellow	Yellow at anode, Orange at cathode	-	[45]
**C7f**	-	580, 472, 479	0.22	Yellow	Blue at anode, Orange at cathode	-	[45]
**C8a**	Ca. 300	591	0.19	Orange	Orange at anode, Green at cathode	-	[46]
**C8b**	Ca. 300	551	0.16	-	-	-	[46]
**C8c**	Ca. 300	493, 522	0.22	-	-	-	[46]
**C8d**	Ca. 300	493, 522	0.34	-	-	-	[46]

^ denotes changes to emission upon undergoing reduction instead of oxidation.

**Table 4:** ELC parameters of EFC-active non-conjugated polymers.

	Neutral State				Oxidised State			Ref.
	λ_abs_ (nm)	λ_em_ (nm)	Φ	Emission Colour	Changes/Effects on Emission	Contrast Ratio I_OFF/ON_	Response Time, *t* (s)	
**P1a**	Ca. 420	Ca. 450	0.56	Blue	Quenched	-	-	[47]
**P1b**	Ca. 420	Ca. 450	0.63	Blue	Quenched	-	-	[47]
**P2**	369	540*	-	Yellowish Green	Quenched	-	<10 s	[48]
**P3**	460	630	-	Red	Partially Quenched	-	-	[49]
**P4a**	367/366 *	581	0.0041	-	-	-	-	[50]
**P4b**	367/365 *	560	0.0037	-	-	-	-	[50]
**P4c**	358/359 *	501	0.0068	-	-	-	-	[50]
**P4d**	361/364 *	525	0.0059	-	-	-	-	[50]
**P4e**	334/331 *	441	0.471	Blue	Quenched	12.7	-	[50]
**P5a**	365/350 *	448	0.092/0.078 *	-	-	-	-	[51]
**P5b**	355/359 *	452	0.257/0.191 *	-	-	-	-	[51]
**P5c**	357/359 *	463	0.329/0.234 *	-	-	-	-	[51]
**P5d**	355/364 *	454	0.341/0.256 *	Blue-green	Quenched	221.4		[51]
**P5e**	366/353 *	492	0.02/0.019 *	-	-	-	-	[51]
**P6**	318, 389/320, 375 *	442	0.502	Blue	Quenched	152	-	[52]
**P7a**	309/312 *	488/474 *	0.262/0.321 *	Cyan	Quenched	234	-	[53]
**P7b**	332/336 *	486/478 *	0.04/0.057 *	Cyan	-	-	-	[53]
**P7c**	338/334 *	485/482 *	0.022/0.027 *	Cyan	-	-	-	[53]
**P8a**	319/318 *	490 *	0.009/0.073 *	Brilliant Blue	Quenched	206	3.1 (off) 1.1 (on)	[54]
**P8b**	315/314 *	514	0.017/0.691 *	Green-Yellow	Quenched	417	6.7 (off) 1.2 (on)	[55]
**P9a**	310/315 *	492/470 *	0.14/0.46 *	Blue	Quenched	98	8.6	[56]
**P9b**	339/343 *	493/510 *	0.03/0.16 *	Green	Quenched	64	7.1	[56]
**P9c**	347/353 *	498/554 *	0.008/0.05 *	Yellow	Quenched	48	6.5	[56]
**P10a**	322/335 *	476/475 *	0.573	Violet	Quenched	-	-	[57]
**P10b**	327/340 *	479/478 *	0.027	Violet	Quenched	-	-	[57]
**P10c**	323/320 *	475/479 *	0.39	-	-	-	-	[57]
**P10d**	342/352 *	478/473 *	0.02	-	-	-	-	[57]
**P11**	316 *	435 *	-	Blue	Quenched	151.9	-	[58]
**P12a**	314/316 *	454/427 *	0.043/0.104 *	-	Quenched	92	-	[59]
**P12b**	307/312 *	456/429 *	0.026/0.061 *	-	-	-	-	[59]
**P13**	-	450	-	-	Quenched	6.67	11.2 (off) 5.2 (on)	[60]
**P14**	330	518	-	Green	Quenched	33.3	9.4 (off) 10.8 (on)	[63]
**P15**	-	406/486 *	-	-	Quenched	4.6	10.5 (off) 9.2 (on)	[61]
**P16**	Ca. 460	484 *	-	Blue	Partially Quenched	2.5	4.2 (off) 2.4 (on)	[62]

* denotes absorption and emission measurements done on solid thin film state.

**Table 5:** ELC parameters of EFC-active conjugated polymers.

	Neutral State				Oxidised State			Ref.
	λ_abs_ (nm)	λ_em_ (nm)	Φ	Emission Colour	Changes/Effects on Emission	Contrast Ratio I_OFF/ON_	Response Time, *t* (s)	
P17	425	535	0.0032; 0.0030	Green	Quenched; Φ = 0.000035; 0.000032	100	-	[67]
P18a	315, 450	580	-	Yellow	Quenched.	19.2	-	[69]
P18b	450/465 *	580/580 *	-	Yellow	Partially Quenched	5	-	[68]
P19a	346/340 *	455/450 *	0.261	Blue	Quenched	-	3.39 (off) 2.88 (on)	[70]
P19b	375/378 *	520/491 *	0.171	Blue	-	-	2.18 (off) 2.08 (on)	[70]
P19c	356/346 *	505/493 *	0.042	Blue	-	-	3.56 (off) 3.80 (on)	[70]
P20a	366/365 *	415/423 *	0.87/0.77 *	Blue	Partially Quenched	-	-	[71]
P20b	360/358 *	432/443 *	0.82/0.73 *	Sky Blue	Quenched	16.3	3	[71]
P20c	386/376 *	434/435 *	0.65/0.43 *	Blue	Quenched	-	-	[71]
P20d	378/358 *	432, 565/565 *	0.22/0.17 *	Yellow	Quenched	21.4	4.22 (off) 4.33 (on)	[72]
P20e	Ca. 375	Ca. 565	0.23	Greenish Yellow	Quenched	23.8	4.54 (off) 5.62 (on)	[73]
P20f	-	Ca. 455	0.74	Blue	Quenched	21.9	4.46 (off) 4.33 (on)	[73]
P21	Ca. 425	Ca. 525	0.038	Yellow	Increased slightly then quenched (Φ = 0.0021)	18	-	[74]
P22	407/417 *	540	-	Yellow-green	Quenched	-	-	[75]
P23a	570/589 *	602/621 *	<0.01	-	-	-	-	[76]
P23b	402/420 *	483/533 *	0.045	Yellow	Quenched	-	-	[76]
P23c	430/445 *	492/573 *	<0.01	-	-	-	-	[76]
P24a	441/452 *	533/547 *	-	Green	Quenched	-	-	[77]
P24b	480/522 *	617/636 *	-	Red	Quenched	-	-	[77]
P25a	416	545	-	Yellow	Quenched	-	2.9 (off) 2.7 (on)	[78]
P25b	412	539	-	Yellow	Quenched	-	2.8 (off) 2.8 (on)	[78]
P25c	418	561	-	Orange	Quenched	-	2.5 (off) 3.0 (on)	[78]
P25d	425	554	-	Orange	Quenched	-	3.0 (off) 2.7 (on)	[78]
P26	364/362 *	473/470 *	0.117/0.219 *	Bluish Green	Quenched	242	<0.4	[79]
P27a	389 *	521 *	0.28	Green	Quenched	21.8	0.19	[80]
P27b	Ca. 340 *, 425 *	Ca. 625 *	0.021	Red	Quenched	11.3	0.92	[80]
P28a	356 *	473 *	-	Blue	Quenched	64	-	[81]
P28b	426 *	582 *	-	Yellow	Quenched	179	-	[81]

*: Absorption and emission data were collected on solid thin film state.
